# Sensing the Deadliest Toxin: Technologies for Botulinum Neurotoxin Detection

**DOI:** 10.3390/toxins2010024

**Published:** 2010-01-07

**Authors:** Petr Čapek, Tobin J. Dickerson

**Affiliations:** 1 Department of Chemistry, The Scripps Research Institute, 10550 North Torrey Pines Road, La Jolla, CA 92037, USA; Email: capek@scripps.edu; 2 Department of Chemistry and Worm Institute for Research and Medicine, The Scripps Research Institute, 10550 North Torrey Pines Road, La Jolla, CA 92037, USA

**Keywords:** botulinum neurotoxin, detection, endopeptidase, botulism, mouse lethality assay, ELISA, lateral flow test, mass spectrometry, FRET, immuno-PCR

## Abstract

Sensitive and rapid detection of botulinum neurotoxins (BoNTs), the most poisonous substances known to date, is essential for studies of medical applications of BoNTs and detection of poisoned food, as well as for response to potential bioterrorist threats. Currently, the most common method of BoNT detection is the mouse bioassay. While this assay is sensitive, it is slow, quite expensive, has limited throughput and requires sacrificing animals. Herein, we discuss and compare recently developed alternative *in vitro* detection methods and assess their ability to supplement or replace the mouse bioassay in the analysis of complex matrix samples.

## 1. Introduction

Botulinum neurotoxins (BoNTs) are the most poisonous substances known to humans, with a median lethal dose (LD_50_) of approximately 1 ng per kg of body weight [1] and are the cause of the life-threatening neuroparalytic disease botulism [2]. BoNT intoxication is presented by flaccid paralysis originating from an inhibition of neuromuscular signal transmission. Lethality of the disease is then connected with muscle paralysis-caused respiratory failure. On a molecular level, BoNTs are zinc-dependent metallo¬proteases that cleave SNARE (soluble *N*-ethylmaleimide-sensitive factor attachment protein receptor) complex proteins that are critical for the release of the neurotransmitter acetylcholine from neuronal cells [3].

BoNTs are produced by the Gram-positive anaerobic soil bacterium *Clostridium botulinum* [4], first discovered as a contaminant of poorly preserved ham in the late 19^th^ century. Besides soil and spoiled food, *C. botulinum* can grow in wounds or the human intestine and can be also cultured in large scale in a laboratory. Due to the extreme potency, lethality and easy procurement, BoNTs have the potential to be very dangerous biological weapon and therefore represent significant warfare and terrorism threat [5]. Consequently, BoNTs are one of the six category A agents listed as the highest risk threat agents for bioterrorism by the US Centers for Disease Control and Prevention (CDC) [6].

Apart from being a dangerous biohazard agent causing incidental death and a potential biological weapon, BoNTs also have important therapeutic value. These toxins are utilized in the treatment of a wide variety of conditions including cervical dystonia, strabismus, blepharospasms, hemifacial spasms, hyperhidrosis, myofacial pain, migraine headaches, vocal cord dysfunction, diabetic neuropathy, anal fissure and multiple sclerosis [7,8,9]. Additionally, the most well-known application of botulinum neurotoxin serotype A (BoNT/A) is its use in the cosmetic industry as an anti-wrinkle agent, under the commercial name Botox^®^.

### 1.1. Molecular Mechanism of BoNT Action

BoNTs are produced by *C. botulinum* as a single 150 kDa inactive protein, which becomes activated by proteolytic cleavage into the light chain (LC) metalloprotease catalytic domain (50 kDa) and heavy chain (HC), which consists of translocation and binding domains (100 kDa) [10]. These two chains are linked as a heterodimer by a single disulfide bond, as well as numerous non-covalent interactions between the two peptide chains. There are seven different serotypes of BoNTs, named A-G; these are up to 70% different at the amino acid sequence level, but all serotypes share similar folded conform­ations and identical activity at the organismal level, albeit with slightly different molecular targets.

**Figure 1:**
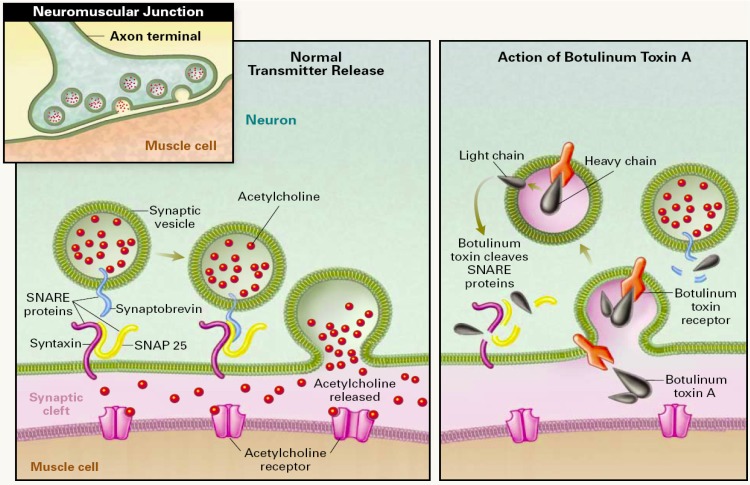
Mechanism of action of botulinum neurotoxin. *Left side:* Release of acetylcholine at the neuromuscular junction is mediated by the assembly of the SNARE protein complex, allowing the the membrane of the synaptic vesicle containing acetylcholine to fuse with the neuronal cell membrane. SNARE protein complex includes synaptobrevin, SNAP-25, and syntaxin. *Right Side:* BoNT binds to the cell membrane and enters the neuron by endocytosis, the light chain is translocated through the membrane and then cleaves specific sites on the SNARE proteins, preventing complete assembly of the synaptic fusion complex and thereby blocking acetylcholine release. Botulinum toxins types B, D, F, and G cleave synaptobrevin; types A, C, and E cleave SNAP-25; and type C cleaves syntaxin. Reprinted with permission from [19]. Copyright © 2002 Massachusetts Medical Society. All rights reserved.

BoNT intoxication occurs in three steps: (i) neuronal cell specific binding and internalization by receptor-mediated endocytosis, (ii) translocation and release of the LC into the cytosol and (iii) cleavage of the SNARE complex proteins (Figure 1) [11]. Examining this process in more detail, toxin binding to neuronal cells occurs via HC binding to two receptors. Toxin first associates with the cell membrane via a ganglioside followed by migration of the complex to its cognate protein receptor [12,13]. Upon binding to both receptors, toxin is then internalized by endocytosis. After endocytosis, LC escapes the endosome through an endosome membrane translocation process [11]. It is believed that as the pH in the endosome lowers, it triggers a subsequent conformation change in the toxin, resulting in the HC acting as a transport channel and chaperone, facilitating LC translocation through the endosome membrane and into the cytosol [14,15]. Finally, inside the cytosol, the LC acts as zinc-dependent metallo¬protease and cleaves proteins of the SNARE complex, which are the part of exocytosis apparatus, effectively destroying this apparatus and leading to inhibition of neurotransmitter release [3]. In this last step of SNARE complex protein cleavage, each of the seven different BoNT serotypes cleaves a unique peptide bond located on one of the SNARE proteins [16,17,18]. BoNT/A, /C and /E cleave synaptosomal associated protein of 25 kDa (SNAP-25), at positions 197-198, 198-199 and 180-181, respectively. BoNT/B, /D, /F and /G target synaptobrevin, cleaving at positions 76-77, 59-60, 59-59 and 81-82, respectively. Interestingly, in addition to SNAP-25, BoNT/C also cleaves syntaxin at position 253-254.

### 1.2. Botulism

Human botulism is caused mainly by BoNT/A, /B, /E and occasionally /F, with BoNT/A being the most poisonous to humans followed by BoNT/B. Ingestion of food contaminated with BoNT-producing *C. botulinum*, wound infections and intestinal colonizing infections in infants (as well as adults with misbalanced intestinal flora) are three most common causes of botulism disease [5,20,21]. In the case of an infected wound or intestine, toxin is produced *in vivo* by *C. botulinum*. The symptoms and manifestation of botulism are identical for all serotypes, with the onset of flaccid paralysis usually occurring within 12 to 48 hours after intoxication. Muscle paralysis typically starts with the facial muscles controlled by cranial nerves, causing double vision, drooping eyelids, *etc.* and continues to descend into shoulders, arms and finally legs. Severe botulism leads to paralysis of respiratory muscles and respiratory failure [22].

Treatment of botulism has to be started as soon as possible after intoxication as paralysis cannot be reversed by any therapeutic intervention. The current standard of care consists of administration of equine antitoxin (commonly antibodies against three serotypes, BoNT/A, /B and /E) which binds to toxin that has not yet internalized into neuronal cells. It is critical to note that antitoxin is unable to enter poisoned cells and reverse the paralysis. Other support such as mechanical ventilation is provided to patients with severe botulism for weeks or months until full recovery [22].

## 2. BoNT Detection

Due to the extreme toxicity, the speed of symptom onset and lack of treatment to reverse paralysis, a sensitive and rapid BoNT detection method is needed to diagnose botulism in suspected cases before paralysis occurs. Additionally, there is a great need for the sensitive detection of BoNT outside of clinical diagnostics. As mentioned above, BoNT represents a high bioterrorism threat. In the case of a terrorist attack, rapid, sensitive and field deployable detection would be needed to assess the extent of contamination and to take necessary action. Finally, with the ever-increasing medical use of BoNT, its sensitive and specific detection in manufacturing processes as well as clinical research laboratories is of crucial importance. Methods of BoNT detection were recently reviewed in several excellent publications [23,24,25]. This article is intended to give the reader an update on the most recent developments and put them into the context of previously reported methods.

## 3. Mouse Lethality Assay

Despite enormous progress in the development of alternative *in vitro* methods for botulinum neurotoxin detection [23,24,25], the mouse lethality assay has remained the only accepted standard test to confirm active BoNTs [26]. This test is based on intraperitoneal injection of mice with dilutions of BoNT-suspected samples and subsequent observation of these mice for symptoms of botulism and, ultimately, death. Such symptoms include fuzzy hair, muscle weakness, narrowed waist, gasping for breath, and subsequent respiratory failure that usually occurs during the first 48 h post injection. It is necessary to find both the maximum sample dilution that kills mice and the minimum dilution that does not kill in order to estimate the quantity of BoNT in the sample. If the dilution that does not kill is not found and all injected animals die, the sample has to be diluted further and the procedure repeated [26]. The quantity of toxin in the sample is then estimated by relating the maximum dilution which kills to the known mouse lethal dose (MLD_50_, 10 pg for BoNT/A) [27]. The toxin serotype is then determined by neutralization of the toxin with serotype specific antitoxin usually administrated prior to toxin injection. Mice are observed for signs of botulism for another 48h to conclude which specific antitoxin is protective. Thus, in order to appropriately conduct this assay, a minimum of four to six days are required before an estimate of toxin concentration can be obtained.

The mouse lethality assay has been used for analysis of complex sample matrices ranging from bacterial cultures to serum, fecal, gastric and wound samples. Examples of matrix interference with the assay are known, particularly when other substances or toxins present in the sample cause lethality [28,29], but the assay is generally considered to be highly sensitive and specific. Another advantage is that it detects functionally active toxin, unlike the majority of immunological methods that do not provide information about toxin activity. However, this is not to imply that the mouse lethality assay is without substantial drawbacks. Several laboratory animals have to be sacrificed for one test, the procedure is laborious and expensive and inherently limited to laboratories with an animal facility. Furthermore, a single test with serotype determination takes a minimum of four days to perform. This is unacceptably long when fast action is needed, for example, in suspected clinical cases of botulism. 

### 3.1. Variations of Mouse Bioassay

Variations of the mouse lethality assay have been developed to reduce the number of laboratory mice required. One variation relates the toxin quantity to the severity of local symptoms (e.g., paralysis), their offset and time-to-death after subcutaneous injection of the sample at the inguinocrural region [30]. This method can estimate BoNT/A quantities ranging from as little as 0.075 mouse LD_50_ to an upper detection limit of about 38 LD_50_s (sensitivity over nearly 3 logs) with just one animal. A similar method based on assessing the severity of flaccid paralysis was developed to analyze low concentrations of therapeutic samples of BoNT/A without the necessity of a terminal end point [31]. In another related method, toxin dilution is injected intravenously and time-to-death is correlated to toxin concentration [32].

More recently, a rat test was developed in which a direct measure of neuromuscular signal transmission, the compound muscle action potential (CMAP), was used to quantify BoNT concentration [33]. The CMAP is generated by the contraction of muscle fiber and changes in the resulting micro current can be directly measured upon toxin treatment. One day post treatment, BoNT/A, C and E could be detected at levels lower than 1 MLD_50_ (with BoNT/A having significant effect on CMAP at levels as low as 0.03 MLD_50_). The related *in vitro* mouse/rat hemidiaphragm muscle contraction assay correlates muscle twitch tensions to the toxin concentration [34]. In this *in vitro* method, hemidiaphragm preparations with attached phrenic nerves are stimulated with a supramaximal pulse and the resulting tension of muscle twitches is recorded. The signal from preparations treated with BoNT is compared to the pre-treatment signal to estimate the toxin concentration. The assay was successfully utilized in a development of BoNT antagonists [35,36].

All of these methods can estimate toxin quantity with significantly fewer animals required than the mouse lethality assay, but the toxin serotype must be known in advance to correlate symptoms and survival times to dose. If the serotype is unknown, a toxin neutralization assay or alternative assay has to be performed to determine which serotype is present. However, all of these mice/rat based assays still have limitations inherently connected to animal experiments, including necessity of an animal facility. In addition, the assays are slow when immediate action is needed, have very limited throughput and are impossible to automate.

## 4. *In Vitro* Methods of BoNT Detection

Alternative *in vitro* methods for detection of BoNT have been developed to overcome some of the limitations of the mouse bioassay [23,24,25,37]. The majority of the *in vitro* assays developed over the last four decades are immunological methods based on the binding of an antibody to the toxin. As such, they are usually easier to perform and are significantly faster; however, unlike mouse bioassays, immunological assays do not discriminate between functionally active and inactive form of the toxin. Early implementation of immunological methods relied on technologies such as radio¬immuno­assays [38,39], passive hemagglutination assays [40], and immunodiffusion assays [41,42,43,44]. Ultimately, these assays were overcome by more sensitive Enzyme-Linked Immunosorbent Assays (ELISA).

## 5. ELISA

Over three decades of use for BoNT detection [45,46,47], ELISA has become by far the most commonly employed method for *in vitro* detection of the toxin [23,24,25]. As a means of detection, ELISA uses specific antibody binding to the toxin. In a typical sandwich ELISA setup, wells of a microtiter plate are first coated with a capture antibody that is specific for the toxin, followed by nonspecific blocking of the remaining well surface by an unrelated protein (e.g., bovine serum albumin, BSA). Then, sample putatively containing the toxin is applied to the well and if present, the toxin binds to the capture antibody, while other components of the sample and matrix are washed out. Subsequently, a detection antibody conjugated to a suitable reporter (e.g., horseradish peroxidase (HRP), alkaline phosphatase) binds specifically to the immobilized toxin. The reporter enzyme then converts a colorless chromogenic substrate (e.g., 3,3’,5,5’-tetramethylbenzidine, TMB, in case of HRP) into a colored product which is spectroscopically quantified using a plate reader. Signal is then compared to a standard calibration curve and the toxin quantity is interpolated. The experiment usually takes 5-6 hours to complete, and is significantly faster than mouse lethality assay. ELISAs with an experimental setup analogous to this were reported to have a wide range of sensitivities, depending on the specific antibody and reporter system used, and have been employed for detection of all BoNT serotypes [48,49,50,51].

Several variations of the ELISA protocol were developed to enhance assay sensitivity. In an amplified ELISA, alkaline phosphatase linked to secondary antibody converts reduced nicotinamide adenine dinucleotide phosphate (NADPH) to reduced nicotinamide adenine dinucleotide (NADH). The resulting NADH then triggers a secondary cyclic enzymatic reaction, that is, the reduction of iodonitrotetrazolium violet (INT-violet) to an intensely colored formazane dye, which can be spectroscopically detected. Amplified ELISA was used for detection of BoNTs with sensitivities ranging from 1-10 MLD_50_[27,52,53]. This technique has been accepted as a method for prescreening the toxin samples prior to the mouse lethality assay [26], and validated for screening of food samples [54]. Another example of an amplification system used to enhance the sensitivity of ELISA is the Enzyme-Linked Coagulation Assay (ELCA) [55,56,57]. ELISA-ELCA relies on a complex multistep amplification cascade starting with cleavage of factor X by snake venom coagulation factor conjugated to a detection antibody and ending with the color change of an alkaline phosphatase substrate. Despite the good sensitivity of this method, which is comparable to or exceeding that of mouse lethality assay ( < 1 MLD_50_), it has not been widely accepted due to its excessive complexity.

Serotype specificity of the ELISA depends on the specificity and cross-reactivity of the antibodies used. All BoNT serotypes are immunogenic and can elicit production of antitoxin antibodies. The seven serotypes differ by up to 70% on the amino acid level, thus making the selection of antibodies with little or no cross-reactivity possible [58]. Both monoclonal and polyclonal antibodies have been used in ELISA experiments for the detection and serotyping of BoNTs, with polyclonals being more common due to reduced procurement costs and easier accessibility. Polyclonal antibodies with high specificity against serotypes A, B, E, and F (serotypes generally causing human disease) were employed to identify these BoNT serotypes by amplified-ELISA (amp-ELISA) and ELISA with digoxigenin-labeled antibodies (DIG-ELISA) [26,27] with high sensitivity in the range of 1-10 MLD_50_ and no cross-reactivity. Like amp-ELISA, DIG-ELISA was developed by the Food and Drug Administration (FDA) for prescreening samples for the presence of BoNTs prior to the mouse lethality assay [26]. Sensitive ELISAs relying on monoclonal antibodies with high serotype specificity have also been developed [48].

ELISA methodologies have been successfully employed in the detection and quantification of purified botulinum toxin [49,51,59], in *C. botulinum* cultures that produce the toxin [60,61,62], in an extensive variety of food samples [52,54,63,64,65] (both contaminated food and food artificially spiked with the toxin), and in clinical samples such as serum [49] and feces [66]. Some foods tend to interfere with ELISAs and decrease their sensitivity; therefore, results should be confirmed by mouse lethality assay. The degree of food sample interference with ELISAs is difficult to anticipate *de novo* and must be examined on an individual basis. However, there are some general considerations to make; for example, protocols employing biotinylated reagents (e.g., antibodies) are not suitable for investigation of samples containing eggs due to the high content of avidin in egg whites. Furthermore, interference of clinical samples of feces with ELISAs may be even more severe than that of food samples [66]. It has been shown that conditions of the assay can be modified appropriately (e.g., with dilution into fetal bovine serum (FBS) buffer) to decrease the amount of the feces sample interference [66].

### 5.1. Alternative ELISA Formats

**Figure 2:**
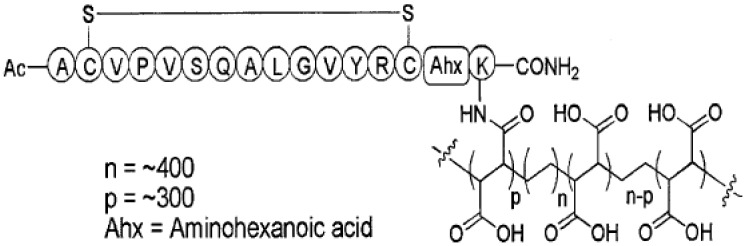
Cyclic peptide identified by phage display conjugated to the polymer used as the capture agent in an ELISA. Reprinted from [67], Copyright © 2006, with permission from Elsevier.

Apart from antibody capture, peptide capture-based ELISAs have also been developed as a sensitive and cheaper alternative as large quantities of peptide can be chemically synthesized, contrary to the significant expense of antibody production [67]. An 11-mer cyclic peptide identified by phage display technology was attached to a polymer matrix and used as capture phase in an ELISA experiment (Figure 2). In this case, the capture peptide polymer showed high specificity for BoNT/A, bringing sensitivity of the assay to a respectable 1 pg/ml of BoNT/A [67].

Another variation introduced into ELISA protocols is the use of ganglioside-bearing liposomes as the detection agent, substituting the detection antibody [68]. Gangliosides are glycosphingolipids present in the membranes of neuronal and other cells known to be receptors for bacterial toxins [69,70], with the trisialoganglioside GT1b characterized as a co-receptor of BoNTs [71,72]. Gangliosides were embedded into the membrane of liposomes together with fluorophore-labeled lipid, and bound to the capture antibody/toxin complex as the detection agent. High theoretical amplification of signal can be achieved due to the fact that every liposome carries tens of thousands of fluorophore molecules. However, the sensitivity of the method for BoNT/A was only 150 ng/ml, presumably owing to unspecific interactions of the liposomes.

**Figure 3:**
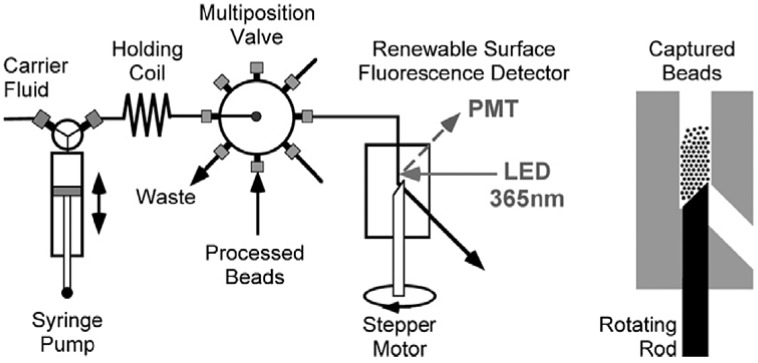
Diagram of the fluidic system with renewable surface flow cell (in detail on the right) used for autonomous BoNT detection. Reprinted from [73], Copyright © 2009, with permission from Elsevier.

Systems for automated ELISAs relying on plate washers and liquid handlers are available to accelerate BoNT detection experiments and reduce the labor requirements. Alternatively, an automated fluidic system consisting of a pump, multichannel selection valve, renewable surface flow cell and on cell detector has been developed to perform sandwich immunoassays (Figure 3) [73,74,75,76]. A given sample solution is applied on beads coated with capture antibody, followed by application of detection antibody and readout by built-in detector, all in an autonomous fashion. Rapid detection (20 min) of BoNT/A heavy chain fragment was achieved with sensitivity around 1 ng/mL [73,74]. While this sensitivity does not compare favorably with ELISA assays, the development of automated and fast detection methodologies remains a topic of interest in the detection of BoNTs.

## 6. Flow Cytometric Assays

Fluorescent sandwich immunoassays have been also performed on beads with flow cytometry instrumentation used to detect and quantify toxin [77,78,79]. Detection of BoNT/A and BoNT/B was performed in multiplex with other toxins (e.g., cholera toxin, ricin and staphylococcal enterotoxin B (SEB)) using different color-coded beads [78,79]. The use of magnetic beads in flow cytometry was shown to have several advantages including that a preconcentration step can be involved, resulting in increased sensitivity and making analysis of turbid or heterogenous samples possible as beads can be easily separated from the matrix [79]. BoNT/A and B were detected at concentrations of 21 ng/mL and 73 ng/mL, respectively, in 5-plex assay together with ricin, SEB and abrin. This sensitivity was comparable to the sensitivity of an ELISA performed for one analyte at the time with the same set of antibodies (detection limits of 12 ng/mL and 124 ng/mL, respectively, for BoNT/A and B). Another assay was performed using an automatic fluidic system format. Beads with capture antibody were trapped in a flow cell, where sample toxin capture, washes, and binding of detection antibody were performed under the control of a system before flow cytometric analysis. The detection limit of this assay was found to be 50 pg/ml of BoNT/A heavy chain (used as nontoxic simulant) [77].

Although being based on the same principle of sandwich immunoassay, flow cytometric assays have several significant benefits over ELISA. Flow cytometric assays are easier to automate and therefore can be less laborious; assays are easier to multiplex and therefore, it is easier to interrogate samples for the presence of multiple toxins and/or several BoNT serotypes in one test tube; there are inherent advantages of capture on beads compared to a well surface including better capture kinetics and enhanced analyte concentration. Additionally, quantitation of toxin in a given sample by flow cytometric assay is faster than ELISA. Also, it is reasonable to expect that the sensitivity of flow cytometric assays will at least reach that of sensitive ELISAs. However, that does not imply that this technique is not without drawbacks. In particular, the instrumentation needed to perform flow cytometric analysis is significantly more expensive and complex than the plate-readers needed for ELISA. 

## 7. Electrochemiluminescence Immunoassay

Electrochemiluminiscence (ECL) immunoassays have been employed in BoNT detection as an alternative to standard ELISAs [80,81,82,83]. In essence, ECL immunoassays rely on the same type of interactions between capture antibody, analyte and reporter antibody involved in the majority of immunoassays. The primary difference is that the reporter antibody in an ECL assay has an electrochemiluminiscence tag, such as a ruthenium (II) tris(bipyridyl) complex, which becomes luminescent in the presence of an electric potential. Unlike ELISA, ECL assays are typically performed on magnetic beads coated with capture antibody. Upon binding of the toxin and reporter antibody, beads are directed by a magnet to an electrode where the ECL reaction occurs. Potential gains in sensitivity can come from several sources: (i) higher luminescent signal-to-noise ratio; (ii) high surface area of beads allowing for denser antibody packing; (iii) enhanced kinetics of antibody-antigen interactions because beads are free in suspension; (iv) volume of sample probed by beads can be greater than the volume of the ECL detection reaction upon concentration of the beads by a magnet, introducing another amplification step.

In an early example of an ECL assay, BoNT/A was detected at concentrations as low as 5 pg/mL [80]. Direct comparison of detection sensitivity of ECL and ELISA using the same antibodies and purified BoNT/B as analyte has been also performed [81]. With a detection limit of about 1 ng/mL, the ECL methodology performed about four-fold better than comparable ELISA in terms of sensitivity and was twice as fast. In another assay, ECL was used for detection of BoNT/A, B, E and F in a limited number of foods and clinical samples [82]. Sensitivity in tested matrices ranged from 50 pg/ml (BoNT/A) to 400 pg/mL. However, despite the above mentioned advantages of ECL, the sensitivity boost compared to ELISA is relatively marginal. Additionally, the number of matrices tested with ECL is very limited compared to ELISAs, and the equipment needed for ECL detection is more specific. Consequently, all of these issues preclude wide spread application of this method.

## 8. Immuno-PCR

The polymerase chain reaction (PCR) enables exponential amplification of a DNA template, making it a powerful tool for sensitive nucleic acid detection. Immuno-PCR is an ELISA-type immunoassay that uses PCR for exponential amplification of ELISA signal [84]. As in the classical sandwich ELISA described above, analyte (BoNT) is captured by the adsorbed antibody and detected by reporter antibody binding to preformed capture antibody/analyte complex. In the case of immuno-PCR, the reporter antibody is a DNA-antibody conjugate with DNA used as an amplifiable tag. Amplification of DNA is performed either by normal PCR, which requires agarose gel electrophoresis detection of the PCR product, or more conveniently, by real-time quantitative PCR, which is capable of direct DNA quantification using fluorescent dye labeling of the formed PCR product.

Immuno-PCR has been used for detection of BoNT/A with sensitivity reaching that of the mouse lethality assay [85]. In another report, sensitivity of as little as 1 pg/mL of BoNT/A in buffer was accomplished, using streptavidin to connect the biotinylated DNA tag to biotinylated antibody instead of covalent DNA-antibody conjugation [86].

**Figure 4:**
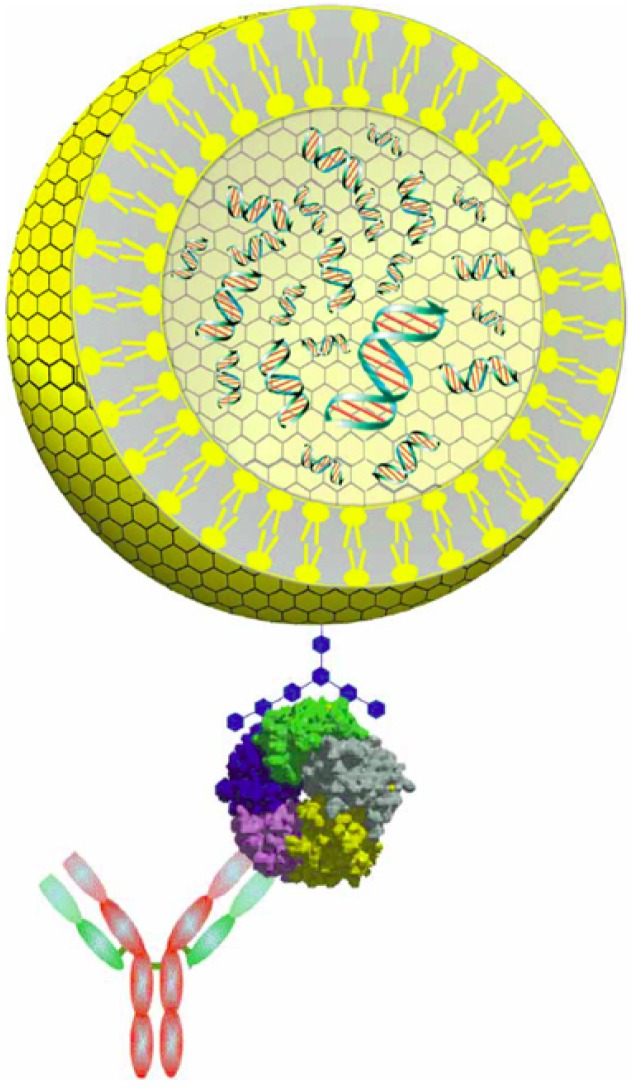
A representation of liposome-PCR reagents. The dsDNA reporter (green with red bars) is encapsulated inside the liposome (yellow) with ganglioside receptor (blue) incorporated into the membrane. Liposome reagent binds to the analyte (shown here as a pentameric protein) which is captured by an antibody. Reprinted by permission from Macmillan Publishers Ltd. from [87]. Copyright © 2006.

An alternative assay termed liposome-PCR has been developed recently to detect BoNT and cholera toxin [87,88]. In this assay, approximately 60 copies of reporter DNA are encapsulated in a liposome, which has its outside surface labeled with ganglioside (trisialoganglioside GT1b for BoNT binding, Figure 4). The surface GT1b of DNA-loaded liposomes then binds to a capture antibody/BoNT complex followed by liposome rupture and real-time quantitative PCR of released DNA. To eliminate contaminating DNA which could be present in the sample and could increase background signal, captured liposomes can be treated with DNase to digest any contaminating DNA outside of the liposomes. By liposome-PCR, BoNT/A at a concentration as low as 0.02 fg/mL was detected in purified water [87], making this method five orders of magnitude more sensitive than the mouse lethality assay and the most sensitive *in vitro* immunological method reported. However, application of this assay in the detection of BoNT in complex matrices was not reported. Nonspecific binding of liposomes, as well as their stability in presence of complex matrices, may be problematic and could be a significant drawback to this method.

## 9. Lateral Flow Tests

**Figure 5:**
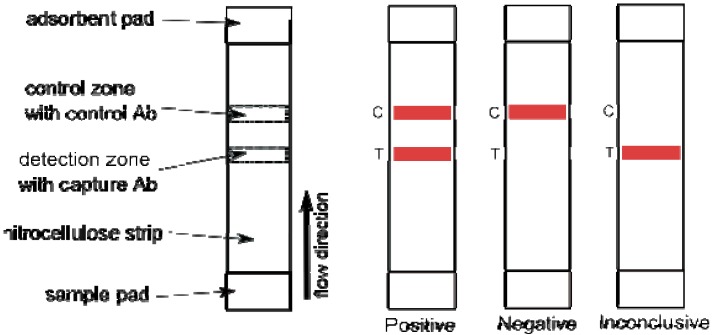
Schematic view of a typical lateral flow device on the left. Three possible results; positive, negative and inconclusive due to missing control signal, are shown on the right.

The lateral flow test is a hand-held immunochromatographic assay that also relies on a capture antibody-analyte-detection antibody interaction, typically on a nitrocellulose strip resulting in visual (color) change of the strip. A good example of this type of detection device is the commercial pregnancy test. In the lateral flow test for BoNT detection, liquid toxin sample is applied on one end of the strip and then migrates towards the opposite end via capillary action. First, toxin binds to a detection agent (antibody) conjugated to reporter (e.g., dye, gold nanoparticles) then the whole complex toxin/detection antibody continues to migrate down to the strip, ultimately to be captured in the detection zone by the capture antibody, resulting in a color change in the detection zone (Figure 5).

Lateral flow tests have significant advantages over other detection methods. They are inexpensive, easy to use, generate a visual read-out with no equipment needed and are very rapid with a typical analysis taking only 15 min. All of these properties make them ideal for field use by untrained personnel. However, the price for this ease of use is lower sensitivity compared to ELISA and other modern immunological methods. Typical limits of detection of lateral flow tests for BoNT employing detection antibody conjugated to gold nanoparticles range from 5 to 50 ng/mL (500-5,000 MLD_50_/mL) [89,90,91,92]. Despite this reduced sensitivity, some of these assays are commercially available [89,91]. Successful detection of BoNT/A, B and E with lateral flow assay in a wide variety of food samples has been demonstrated, showing the importance of sample preparation and pre-treatment to increase assay reliability [89].

A system using a cross-flow immunochromatographic method was developed to enhance the sensitivity of the lateral flow BoNT assay [93]. This assay relies upon a detection antibody conjugated to HRP, instead of the more common gold nanoparticles or colloidal gold reporters. After application of the sample and development in one direction (vertical direction flow) as with a regular lateral flow assay, a HRP substrate is applied to the side and the strip is developed in the direction perpendicular to the first one (horizontal direction flow). The HRP chromogenic substrate migrates over the HRP enzyme in the capture zone and its cleavage translates into a color change, signaling presence of the toxin. The sensitivity of this assay is 2 ng/mL (200 MLD_50_/ml) of BoNT/A [93]; a modest enhancement compared to gold nanoparticle-based devices is paid for by increased complexity of the device and the assay protocol. A more effective and simpler signal enhancement procedure using silver enhancer has been developed [94]. In this application, upon development, a lateral flow device employing colloidal gold conjugated reporter antibody was soaked in silver enhancer for 5 min followed by fixing for 3 min. This procedure increased sensitivity of the method 1,000-fold from 50 ng/mL of BoNT/B to 50 pg/mL.

A lateral flow assay with significantly improved sensitivity has also been reported that uses ganglioside-liposomes as the detection agent [95]. Trisialoganglioside GT1b was incorporated by means of its fatty tails into liposomes containing sulforhodamine B dye as a visualization agent to form stable ganglioside-liposomes. These liposomes were used as the detection agent in a lateral flow device and upon binding to the BoNT/A, migrated to reach the detection zone. There, the toxin/ganglioside-liposome complex was retained by the capture antibody, forming a macroscopically visible band. The detection limit of this device was reported to be an impressive 15 pg/mL (1.5 MLD_50_/mL) of BoNT/A in buffer [95]. However, successful application of this method to the detection of the toxin in complex matrices has not been demonstrated.

## 10. Biosensors

Fluorescence-based biosensors are an emerging type of automated portable devices for rapid detection of BoNTs and other biohazard materials [96,97]. Typically, the fluorescence assay these devices use to detect analyte is based on interrogation of the analyte by evanescence wave technology. Surface-bound molecules labeled with fluorophore are excited by an evanescent field, producing a fluorescent signal. A sandwich immunoassay format, consisting of capture antibody, analyte and labeled detection antibody, is used on the surface of the sensor. Since the penetration depth of the evanescent field is limited, only surface bound fluorophores are detected, thus enabling analysis in heterogeneous and turbid samples. Additionally, capture antibodies are easy to array on the detector, allowing for multiplexed detection of bacterial and other toxins.

Using this system, multiple biosensors were tested with BoNTs [98,99,100,101,102,103]. Sensitivities of the sensors range from 150 pg/mL (15 MLD_50_/mL) for BoNT/E [100] to 200 ng/mL for BoNT/B [101] in buffers. Besides single analyte sensors, array sensors for multiple analytes were constructed for the simultaneous detection of several toxins such as BoNT/A, /B, cholera, ricin and staphylococcal endotoxin B, and bacterial samples [101,102]. Detection of BoNT/A toxoid in various food samples has been reported as well with sensitivities typically around 50 ng/mL [103]. Biosensor assays take only 10-20 minutes to complete, regardless of the number of analytes to be detected simultaneously. This makes it one of the fastest assays reported to date. But as in case of lateral flow devices, limited analytical sensitivity is the trade off for the speed.

Recently other biosensors relying on standard fluorescence assays [104,105] or optical immuno-assays [106] have been reported. Interestingly, in one of the assays, the capture antibody was replaced by antimicrobial peptides with broad specificity for not only bacterial pathogens but also for bacterial toxins [105].

## 11. Endopeptidase Activity Based Assays

BoNTs are Zn^2+^-dependent endopeptidases that inhibit neurotransmitter release by specific cleavage of synaptic SNARE complex proteins. Exploitation of this endopeptidase role of BoNTs has led to numerous detection methods. Unlike the immunological *in vitro* methods described above which are unable to discriminate between an active and inactive form of the toxin, endopeptidase assays detect the active form only. For example, if food that was heated tested positive for the presence of BoNT by immunoassay, it may be negative by the mouse lethality assay and endopeptidase assays as the toxin may be inactive. In this sense, endopeptidase assays are closer to the mouse lethality bioassay than immuno¬assays. Detection of the active form is arguably more relevant because only the active form results in the associated morbidity and mortality.

Another advantage of endopeptidase assays is the inherent amplification of the signal by the catalytic cleavage reaction. In an endopeptidase activity assay, the actual analyte is the specific cleavage product rather than the toxin itself. The toxin concentration is then proportional to the concentration of the cleavage product. Because the cleavage process is catalytic and one molecule of the toxin can specifically cleave a large number of substrate molecules, an inherent amplification step is involved. This applies regardless of the actual method used for the detection of cleavage product.

On the other hand, endopeptidase assays are inherently more sensitive to sample matrix interference since toxin catalyzed cleavage of substrate is generally significantly less efficient in complex matrices. To overcome this problem, in some methods, toxin is captured from the matrix first, followed by performing the cleavage reaction in an optimized reaction buffer (see below).

## 12. Fluorescence Endopeptidase Assays

A change in fluorescence of the substrate upon cleavage is often used to detect the endopeptidase activity of an enzyme. High-throughput fluorescence endopeptidase assays have been developed for detection of BoNT/A, /B, /E and /F. In this assay, a serotype-specific peptidic substrate labeled with fluorescein was chemically synthesized and immobilized on a solid support. After specific cleavage with one of the BoNTs, the peptide fragment labeled with fluorescein was released into the solution and spatially separated from uncleaved substrate for quantification [107]. This assay allowed detection of all four serotypes relevant to human botulism at concentrations as low as 2 ng/mL in microtiter plate format.

**Figure 6:**
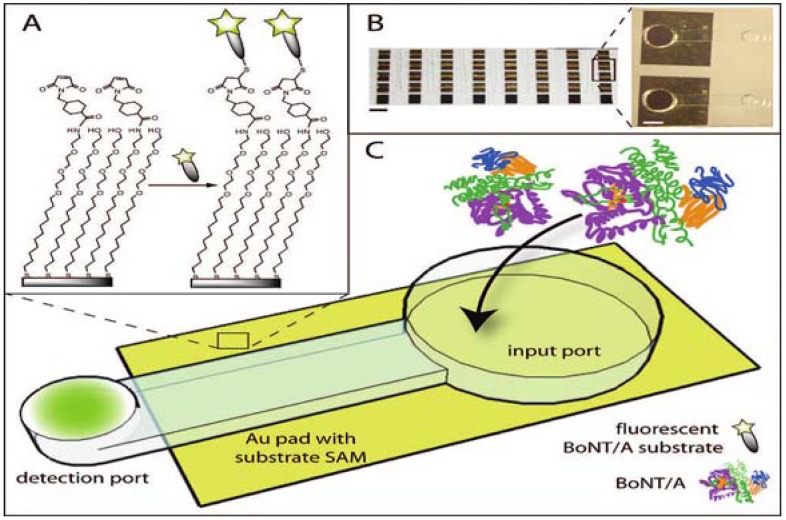
Microfluidic device for fluorescent endopeptidase assay. (A) Fluorescein labeled BoNT/A substrate attached through the linker to the gold surface. (B) View of the array of 40 devices (scale bar = 5 mm). (C) BoNT/A is added into input port. During incubation, immobilized substrate is cleaved and the fluorescent fragment is released into the solution and concentrated at the detection port via evaporation. Reprinted with permission from [108]. Copyright © 2009 American Chemical Society.

More recently, an assay based on the same principle (e.g., cleavage of a fluorophore labeled peptide from a solid support), but working in semiautomatic microfluidic format has been developed [108,109]. The microfluidic device (Figure 6) consists of two ports (input and detection) interconnected by a microchannel. The toxin sample is applied into the input port to catalyze the cleavage reaction of the fluorescein labeled peptide derived from sequence of SNAP-25 from the solid support. The cleaved fluorescein labeled fragment diffuses into the detection port designed to facilitate evaporation of the solution and effectively preconcentrate analyte before fluorescence detection. This evaporation led to 3-fold signal amplification over 35 minutes. The first generation device [109] used a fluorescent substrate tethered to silica beads but both intra- and inter-assay variation in bead load led to a decrease in sensitivity. In an improved second generation device [108] the substrate was tethered to a self-assembled monolayer on a gold surface and this device was able to detect as little as 3 pg/mL of BoNT/A (holotoxin) in buffer. Unfortunately, when tested in a complex matrix, the sensitivity dramatically decreased to 500 ng/mL of BoNT/A (holotoxin), suggesting that use for detection oftoxin in relevant matrices could be limited.

## 13. FRET Endopeptidase Assays

Another widely used technology for the detection of cleavage of peptide bonds is fluorescence resonance energy transfer (FRET). In one setup used for detection of BoNTs, an oligopeptide resembling natural substrate carries two tags flanking the cleavage site. One tag is the so called fluorescence quencher and other is a fluorescence donor. If they are close to each other in space, as in an uncleaved substrate, the fluorescence of an excited donor fluorophore is quenched (absorbed) by the quencher. However, once the substrate is cleaved and the quencher and donor fluorophore become separated, fluorescence of the donor is no longer quenched and can therefore be detected. Multiple variations of fluorophore/quencher modified substrates derived from SNAP-25 have been developed and used for the detection of BoNT/A [110,111,112,113,114,115]. Similarly, internally quenched fluorescent substrates derived from synaptobrevin and syntaxin have been developed [115]. The detection limit of direct FRET based assays depends on the particular FRET substrate used and range from 40 ng/ml of BoNT/A in apple juice or buffer [110,111] to 60 pg of BoNT/A in buffer [113]. As in the case of other endopeptidase assays, food samples tested can severely interfere with the assay [110].

Alternatively, recombinant SNAP-25 or synaptobrevin with cyan fluorescent protein (CFP) and yellow fluorescent protein (YFP) at each side of the protein were employed as FRET substrates instead of chemically synthesized substrates [116,117,118]. The FRET between CFP/YFP pair is detected in the uncleaved protein but fluorescence is abolished once the substrate is cleaved. This CFP/YFP-SNAP-25 sensor could detect 2 ng/mL of BoNT/A and /E *in vitro*; a synaptobrevin based sensor detected approximately 30 ng/mL of BoNT/B and /F *in vitro* within 4 h [116,117]. To further improve assay sensitivity, recombinant protein derived from SNAP-25 having one CFP donor in the middle of two substrate proteins with two YFP acceptors on each side was constructed, enhancing the capture of CFP emission in uncleaved protein and improving *in vitro* sensitivity to 0.3 ng/mL of BoNT/A [118]. Additionally, CFP/YFP sensors were transfected into PC12 cells and the toxin could be detected in living cells [116,117].

### 13.1. FRET Assays with Immunocapture

**Figure 7:**
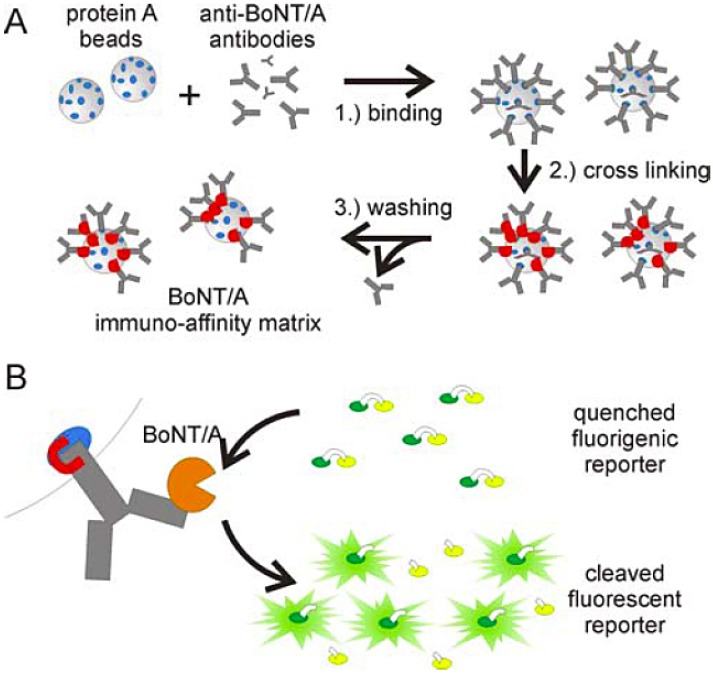
(A) Diagram for the preparation of an immuno-affinity matrix for BoNT enrichment. Protein A beads are coupled to anti-BoNT antibodies and crosslinked via the Fc domain with disuccinimidyl suberate. Non-crosslinked antibodies are washed out. (B) Immobilized BoNT/A cleaves FRET substrate restoring fluorescence. Reprinted from [112], Copyright © 2008 the authors.

Crucial enhancement in the sensitivity of FRET based endopeptidase assays as well as a significant reduction of sample matrix interference was achieved by coupling the assay with an immunoseparation step (Figure 7) [112,114]. In this setup, the toxin is first captured from the sample by beads with toxin specific antibodies. The beads with captured toxin are subsequently resuspended in endopeptidase reaction buffer containing the synthetic FRET substrate to initiate the cleavage reaction. Importantly, the capture antibody utilized must be selected so as not to interfere with the catalytic function of bound BoNT. This system has several advantages: (i) the immunoseparation step eliminates/decreases interference from the matrix by separating the toxin from other sample containing nonspecific proteases, (ii) beads with toxin are resuspended in an optimal cleavage buffer, considerably increasing the efficiency of the cleavage reaction, (iii) toxin can be effectively concentrated by minimizing the volume of the cleavage reaction. This immunoseparation coupled FRET assay has been reported to have the impressive sensitivity of about 1 fg/mL (BoNT/A complex in buffer) which is five orders of magnitude more sensitive than the mouse lethality assay [112]. Comparable sensitivity in a limited number of matrices (e.g., serum, carrot juice, milk) has also been demonstrated. It is clearly the most sensitive assay reported, and only its application to more complex matrices will show if it is robust enough for widespread implementation.

## 14. Immunodetection of Cleavage Product

Immunological detection of proteolytically generated SNARE protein (or synthetic analogue) cleavage products has been used in several assays. Typically, a chemically synthesized peptide substrate, derived from one of the SNARE protein sequences, is immobilized on a solid support and then treated with the toxin. The resulting cleavage product on the support is subsequently detected by cleavage product specific antibodies. Cleavage of SNAP-25 derived substrates was used for the detection of BoNT/A [119,120,121], BoNT/C1 [122], and BoNT/E [120]; cleavage of synaptobrevin has also been utilized for the detection of BoNT/B and its light chain [119,123]. Detection limits of this type of assay range from 200 pg/mL (BoNT/B) [119] to as low as 40 fg/mL (BoNT/A) [120]. However, all detections were performed in optimized reaction buffers. Detection of the toxin in relevant matrices was not demonstrated, but a significant decrease in sensitivity can be expected due to suboptimal cleavage conditions. It has also been demonstrated that the antibodies involved are able to adequately discriminate among the serotype specific cleavage products, suggesting that the assay can be used as a highly specific *in vitro* serotyping method [122].

To overcome matrix interference with the endopeptidase assay, immunoseparation of BoNT/B from the matrix, analogous to that employed in FRET assays, was developed [124]. BoNT/B sample was first applied onto an immunoaffinity column and the matrix was washed out after a short incubation. Biotin-modified substrate in reaction buffer was then applied onto the column and incubated to allow the cleavage to occur. Cleavage product specific antibodies were used for detection of the product upon elution from the column and immobilization on a streptavidin-coated plate. Using this system, 1 MLD_50_ of BoNT/B was dectectable in a limited number of food samples [124].

In another report, BoNT/A1, /A2, /A3, /B1 and /F were captured from media by brain synaptosomes before incubation with an appropriate substrate. Serotype specific cleavage products were detected by ELISA with sensitivities from 0.5 to 10 MLD_50_/mL in buffer or diluted sera and with limited sensitivity in food [125]. The method has its disadvantages: rat brain isolated synaptosomes are not a readily available reagent and differences in sensitivity with and without capture were not clearly demonstrated. However, in this report, synaptosome binding is more of a selection for holotoxin with the correct fold than a separation procedure in that only toxin with functional heavy chain binds. This ability to discriminate to some extent between functional light chain only and functional holotoxin makes this method unique among *in vitro* assays.

## 15. Endopep-MS

A method using mass spectrometry (MS) for the detection of the SNARE cleavage products has been developed at the US CDC; the assay has been called Endopep-MS assay [126,127]. Mass spectrometry in tandem with liquid chromatography can be used not only for the quantification of the toxin, but it is also able to differentiate all seven toxin serotypes by discriminating between each serotype’s unique cleavage product(s). The detection limits achieved a range from 0.039 to 0.625 MLD_50_/mL for BoNT/A, /B, /E and /F in a buffer [126]. However, the sensitivity of the method in serum and stool samples was not as impressive. To enhance the sensitivity of the assay in these and other complex matrices, an antibody capture step for toxin concentration, as well as separation from other nonspecific proteases from the matrix, was introduced [128,129]. Separation/concentration increased the sensitivity of the method by up to two orders of magnitude in reaction buffer and allowed detection of BoNT/A, /B, /E, and /F with sensitivities of 0.1-10 MLD_50_/mL in sera samples and 0.5-100 MLD_50_/mL in stool samples [128]. In order to further expand the identification of serotypes to the identification of subtypes, the authors analyzed samples by means of mass spectrometry after a trypsin digest [130]. As identification of the subtype requires the analysis of the toxin protein itself, significantly more material is needed for the assay (about 2 μg of the toxin).

The endopep-MS assay combined with antibody capture represents a very sensitive detection platform, with the additional advantage of serotype identification in one experiment. Together with the immunodetection of the cleavage product, these are the only two procedures able to detect the active toxin and discriminate between serotypes in a single experiment. However, a liquid chromatography system coupled with a mass spectromether is not available in every laboratory and is certainly less common than the simple plate readers required for quantification of signal from an immunodetection experiment.

## 16. Emerging in Vitro Assays and Technologies

Aptamers are single stranded nucleic acids (DNA or RNA) selected from large nucleic acid libraries and used to bind specific targets such as small molecules or proteins, making them suitable for analytical and other applications [131]. Like antibodies, aptamers have a defined three dimensional structure that allows for specific interaction with the target. Additionally, they are faster and easier to select from a library of binders compared to immunoreagents, and once the sequence of aptamer is known, it can be easily chemically synthesized in high quantities.

**Figure 8:**
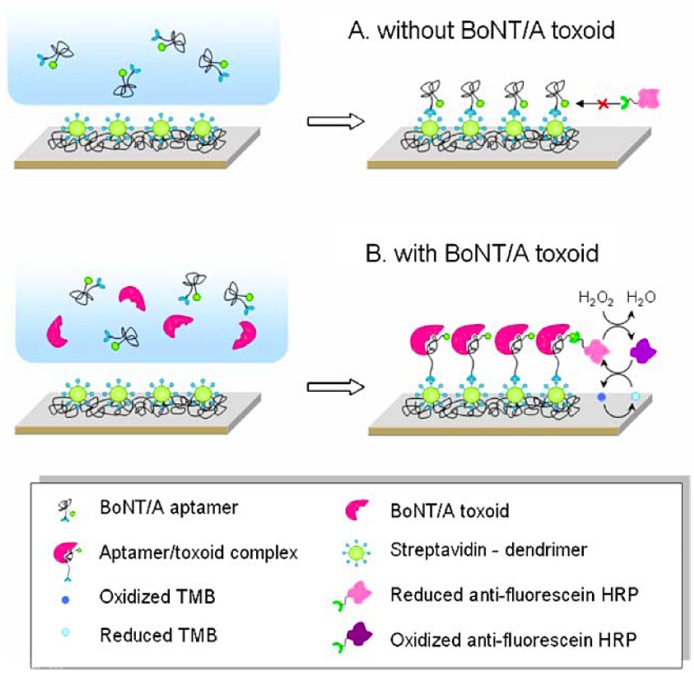
Aptamer-based electrochemical detection of BoNT/A toxoid. Reprinted from [132] with kind permission from Springer Science+Business Media. Copyright © 2009 the authors.

BoNT/A detection methods based on structural changes of an aptamer upon binding to its target have been reported [132,133]. In one of the two reported methods [132], upon binding of BoNT/A to the aptamer, the aptamer can no longer assume the unbound three-dimensional conformation and is accessible to capture conjugated HRP used for amplification of the signal (Figure 8). The assay was able to detect toxoid at a concentration as low as 40 pg/mL in buffer. Unfortunately, the ionic composition of the detection buffer, and in particular, the potassium ion concentration present in the buffer had an enormous impact on assay performance. This can be attributed to the fact that the unbound aptamer contains a long G-quadruplex that is known to be stabilized by potassium ions. 

Microfluidic technology with chemical reactions, biochemical assays and biological processes performed on a chip on the micrometer scale has attracted enormous attention in the last decade. Indeed, detection of BoNT on microfluidic chips has been developed [134,135,136]. Assays carried out on a chip are miniaturized versions of the macroscopic assays described above, such as the sandwich type immunoassay [136] or FRET based endopeptidase assay [134,135]. There are clear advantages to microfluidic technology, including a reduction of reagent consumption and the ability to operate in a semiautomatic mode for all steps (e.g., sample preparation, pretreatment, mixing, incubation, washing) carried out on a chip. Particularly in the context of field deployment, there is huge promise to this technology. 

In addition, there are various other techniques emerging as potential BoNT detection platforms. For example, surface plasmon resonance [137,138,139], rapid detection by transistors with gates modified by anti-toxin antibodies by change in drain-source current upon BoNT binding [140], liquid chromatography coupled to mass spectrometry [141], capillary electrophoresis [142], micro¬mechano­sensor able to detect synaptobrevin molecule cleavage by BoNT/B [143] or time resolved fluorescence assay [144]. Many of these techniques show promise but rely on expensive instrument¬ation and/or have not been sufficiently tested.

## 17. Cell Based Assays

Cell-based assays can be particularly valuable as diagnostics as they can recapitulate the natural environment experienced by an analyte more accurately, particularly when the analyte has a multistep activation mechanism, such as BoNTs. A FRET based cell assay (see above) has been developed [116,117], but this is not the only reported cell-based BoNT diagnostic. Another cell-based assay with fluorescence read-out has been reported recently [145]. Another interesting cell based assay is based on neural cultures grown on microelectrode arrays, which are used to record action potentials of the neural culture [146]. Toxin added into cell media then causes the changes in action potential signature of the culture. Cell based assays are good model for the study of all aspects of BoNT activity: cell surface receptor binding, endocytosis internalization, membrane translocation and finally SNARE cleavage. While *in vitro* activity assays only detect endopeptidase activity, the cleavage of the SNARE proteins in a cell occurs as the last step of this cascade. This makes these cell based assays and the others [147] ideal for BoNT inhibitor screens. However, their sensitivity in comparison to both mouse assay and *in vitro* assays is limited (usually in ng/mL range) and it is difficult to obtain quantitative results. Furthermore, compared to *in vitro* methods, cell based assays are slow (days for detection), experimentally more demanding and require the maintenance of cell cultures. All of this makes them less suitable for routine toxin detection.

## 18. Summary and Outlook

In recent years, the development of *in vitro* assays for BoNT detection has accelerated, decreasing the limit of detection to femtogram per milliliter range (Table 1). However, none of the assays developed to date has been validated to be robust enough to completely replace the mouse lethality assay, the FDA-approved standard of BoNT detection. Although there are assays having sufficient sensitivity and specificity in simple buffer systems, these characteristics dramatically change when these assays are employed in BoNT detection in complex matrices such as food and clinical samples.

**Table 1:** Summary of BoNT detection techniques.

**Method**	**Detection limit**	**Analysis time**	**Multiplex**	**Automation**	**Matrix interference**
**Mouse lethality assay**	1 MLD_50_(20 pg/mL BoNT/A)	4-6 days	no	no	low
**ELISA**	5 pg/mL-2 ng/mL	5-6 hours	limited	limited	manageable
**Flow cytometric assay**	50 pg/mL-20 ng/mL	4 hours	yes	yes	manageable
**Electrochemiluminescence immunoassay**	5 pg/mL - 50 ng/mL	1 hour	limited	limited	manageable
**Immuno-PCR**	1-5 pg/mL	6-9 hours	limited	no	manageable
**Liposome-PCR**	0.02 fg/mL	7-9 hours	limited	no	N/A
**Lateral flow test**	5-50 ng/mL	15 min	no	-	high
**Biosensor (evanescence wave based)**	150 pg/mL-200 ng/mL	10 min	yes	yes	low
**Fluorescence endopeptidase assay**	3 pg/mL	3 hours	no	on-chip	high
**FRET endopeptidase assay**	60 pg/mL-40 ng/mL	3 hours	no	no	high
**FRET endopeptidase assay with immunoseparation**	1 fg/mL	2.5 hours	no	no	low
**Immuno-detection of cleavage product**	40 fg/mL-200 pg/mL	6 hours	limited	no	high
**Endopep-MS**	0.4-6 pg/mL	3-4 hours	yes	yes	high
**Cell based assay**	1-10 ng/mL	2-3 days	no	no	low

If one was to postulate the characteristics of an “ideal” BoNT diagnostic, a number of features can be readily identified. It is clear that this assay must have at least the sensitivity of the mouse lethality assay, have minimal interference with complex matrices, have suitable throughput and be fast (e.g., performed in less than 1 h). The need for a rapid assay is particularly important in cases of suspected clinical botulism, when fast action is needed to fight disease development. In this scenario, even the mouse lethality assay is not rapid enough. A successful assay would also allow for toxin serotype identification in the same experiment as toxin quantification, such as some types of immunoassays using serotype specific antibodies, endopep-MS or assays with immunodetection of the toxin cleavage product. Multiplexed identification of several toxins simultaneously with their serotypes and full automation of the assay is a further desirable extension beyond this. Additionally, an assay that can distinguish active and inactive forms of the toxin and quantify both would be advantageous since depending on the circumstances, detection of one form over the other may be beneficial. For example, activity of the toxin in older samples can diminish and give false negative results in activity assays, including the mouse lethality assay. On the other hand, as only active toxin can cause botulism, detection based on activity is of great clinical importance. It is likely that to get information about both active and inactive toxin, two independent assays will be needed. Lastly, a technology compatible with a hand-held type of device, or at least portable device, is essential for field deployment as in the scenario of a response to a bioterrorist attack.

These requirements are difficult to match and even the mouse lethality assay that has been used for decades does not fulfill all of the requirements of an “ideal” assay. To eliminate or minimize the effect of the matrix on assay performance has proven quite difficult in a number of technologies; in order to match this requirement, a given *in vitro* assay must perform with minimal variation under a wide variety of suboptimal conditions associated with a large range of sample matrices. Clearly, in all cases using the current technology available for BoNT detection, more research is required to validate the methods with a variety of complex matrices.

Tremendous progress has been made in the development of BoNT diagnostics and while the developed assays to date may not yet be fully validated, a set of capable alternatives to the mouse lethality assay are now available. While there is no single method that fulfills all the requirements of an ideal assay, individual methods have been developed that can address varying aspects of these requirements, allowing one to find the right assay for a given scenario.

## Supplementary Files

Supplementary File 1: PDF-Document (PDF, 73 KB)
