# Correction: Čapek, P., *et al.* Sensing the Deadliest Toxin: Technologies for Botulinum Neurotoxin Detection. *Toxins* 2010, *2*, 24-53

**DOI:** 10.3390/toxins2010093

**Published:** 2010-01-19

**Authors:** Petr Čapek, Tobin J. Dickerson

**Affiliations:** 1 Department of Chemistry, The Scripps Research Institute, 10550 North Torrey Pines Road, La Jolla, CA 92037, USA; Email: capek@scripps.edu; 2 Department of Chemistry and Worm Institute for Research and Medicine, The Scripps Research Institute, 10550 North Torrey Pines Road, La Jolla, CA 92037, USA

We realized that in our paper published recently in *Toxins* [1] limits of detection (LOD) of multiplexed fluorescent magnetic suspension assay [2] were incorrectly reported to be in the ng/mL range. Indeed, LODs of this assay are in the pg/mL range and the fourth and fifth sentence in Flow Cytometric Assay section (page 32) should read as follows:

BoNT/A and B were detected at concentrations of 21 pg/mL and 73 pg/mL, respectively, in 5-plex assay together with ricin, SEB and abrin. This sensitivity was comparable to the sensitivity of an ELISA performed for one analyte at the time with the same set of antibodies (detection limits of 12 pg/mL and 124 pg/mL, respectively, for BoNT/A and B).
