# Supplementary material for: Sensing the Deadliest Toxin: Technologies for Botulinum Neurotoxin Detection
Source: Toxins (Basel). 2010 Jan 7;2(1):24–53. doi: 10.3390/toxins2010024 (PMC3206617; doi:10.3390/toxins2010024)
Supplement: Supplementary File 1: — PDF-Document (PDF, 73 KB) [file toxins-02-00024-s001.pdf]

Toxins **2010**, *2*, 93-94; doi:10.3390/toxins2010093

OPEN ACCESS

toxins

ISSN 2072-6651

www.mdpi.com/journal/toxins

Correction

Correction: Čapek, P., *et al.* Sensing the Deadliest Toxin: Technologies for Botulinum Neurotoxin Detection. *Toxins* 2010, *2*, 24-53

Petr Čapek [1] and Tobin J. Dickerson [2,*]

[1] Department of Chemistry, The Scripps Research Institute, 10550 North Torrey Pines Road, La Jolla, CA 92037, USA; E-Mail: capek@scripps.edu

[2] Department of Chemistry and Worm Institute for Research and Medicine, The Scripps Research Institute, 10550 North Torrey Pines Road, La Jolla, CA 92037, USA

* Author to whom correspondence should be addressed; E-Mail: tobin@scripps.edu; Tel.: +1-858-784-2522; Fax: +1-858-784-2590.

Received: 19 January 2010 / Published: 19 January 2010

We realized that in our paper published recently in *Toxins* [1] limits of detection (LOD) of multiplexed fluorescent magnetic suspension assay [2] were incorrectly reported to be in the ng/mL range. Indeed, LODs of this assay are in the pg/mL range and the fourth and fifth sentence in Flow Cytometric Assay section (page 32) should read as follows:

BoNT/A and B were detected at concentrations of 21 pg/mL and 73 pg/mL, respectively, in 5-plex assay together with ricin, SEB and abrin. This sensitivity was comparable to the sensitivity of an ELISA performed for one analyte at the time with the same set of antibodies (detection limits of 12 pg/mL and 124 pg/mL, respectively, for BoNT/A and B).

References

1. Čapek, P.; Dickerson, T.J. Sensing the Deadliest Toxin: Technologies for Botulinum Neurotoxin Detection. *Toxins* **2010**, *2*, 24–53.
2. Pauly, D.; Kirchner, S.; Stoermann, B.; Schreiber, T.; Kaulfuss, S.; Schade, R.; Zbinden, R.; Avondet, M.A.; Dorner, M.B.; Dorner, B.G. Simultaneous quantification of five bacterial and

plant toxins from complex matrices using a multiplexed fluorescent magnetic suspension assay. *Analyst* **2009**, *134*, 2028–2039.

© 2010 by the authors; licensee Molecular Diversity Preservation International, Basel, Switzerland. This article is an open-access article distributed under the terms and conditions of the Creative Commons Attribution license (http://creativecommons.org/licenses/by/3.0/).
